# Optimisation of Heated Electrospray Ionisation Parameters to Minimise In‐Source Generated Impurities in the Analysis of Oligonucleotide Therapeutics

**DOI:** 10.1002/rcm.10033

**Published:** 2025-04-03

**Authors:** Mollie A. Glenister, Ulrik Mistarz, Ken Cook, James L. Stephenson, Mark J. Dickman

**Affiliations:** ^1^ School of Chemical, Materials and Biological Engineering University of Sheffield Sheffield UK; ^2^ ThermoFisher Scientific Allerød Denmark; ^3^ ThermoFisher Scientific Hemel Hempstead UK; ^4^ ThermoFisher Scientific Lexington Massachusetts USA

**Keywords:** electrospray ionisation, ion‐pair reversed phase, oligonucleotide therapeutics

## Abstract

**Rationale:**

Oligonucleotides have emerged as an important new class of therapeutic. Due to their structural complexity, this presents significant challenges for the development of analytical methods to characterise and determine their impurity profile. In this study, we introduce a sensitive ion‐pair reverse phase method interfaced with mass spectrometry for analysis of antisense oligonucleotides and small interfering RNAs.

**Methods:**

Liquid chromatography–mass spectrometry analysis of antisense oligonucleotides and small interfering RNAs was performed using hexylamine: hexafluoro‐2‐propanol mobiles phases. LC‐MS analysis was performed in both negative and positive ion mode. Electrospray ionisation source conditions including collision energy and temperature were optimised to minimise in‐source generated impurities and alkylamine adducts in the analysis of oligonucleotide therapeutics.

**Results:**

The results show that under low or no in‐source collision energy the presence of hexylamine adducts are observed and are predominantly on the lowest charge states present. As the in‐source collision energy is increased, a reduction of hexylamine adducts is observed in conjunction with an increase in nucleobase loss in the gas phase, therefore generating in‐source impurities. In comparison to tributylammonium acetate, increased MS sensitivity, higher charge states and effective removal of hexylamine adducts using mild source conditions was achieved.

**Conclusions:**

Optimisation of the mild source conditions in conjunction with high pH mobile phases was combined with high‐resolution accurate mass spectrometry analysis and automated deconvolution workflows to develop a simplified and streamlined approach for characterising oligonucleotide therapeutics and their related impurities.

## 1 Introduction

Oligonucleotide (OGN) therapeutics are an emerging class of drugs which have the potential to treat a wide range of diseases and conditions, including cancer and neurological conditions [1, 2, 3]. Twenty oligonucleotide therapeutics have been approved by both the Food and Drug Administration (FDA) and the European Medical Agency (EMA) as of March 2024 [4], showcasing the growing interest in this field.

There are a number of different classes of OGN therapeutics including antisense OGNs (ASOs), small interfering RNAs (siRNAs), involved in RNA interference (RNAi), small activating RNAs (saRNAs), aptamers and guide RNAs (gRNAs) involved in CRISPR gene editing technology. The most dominant of these subgroups is ASOs which represent 65% of OGNs having obtained marketing authorisation [5]. This class of OGN therapeutic are single‐stranded nucleic acids which have complementary sequences to target mRNA and results in degradation and the downregulation of specific genes [6, 7]. Modifications to OGN structure are often included in the therapeutic design to improve stability by preventing degradation by endonucleases, increasing cellular uptake and improving affinity to target strands [8].

Recent advances in nucleic acid chemistry have substantially influenced the design and optimisation of OGN therapies and their delivery mechanisms. Chemical stabilisation techniques have been used to enhance pharmacokinetic and pharmacodynamic responses. Chemical modifications are introduced into the structure of the nucleic acids or linkers and typically include replacing the non‐bridging oxygen atoms in the phosphate backbone with sulphur groups (phosphorothioate modifications), which reduces enzyme degradation and increases uptake into cells [9, 10]. Adding an electron‐withdrawing group to the 2′ hydroxyl group of the ribose sugar, including methyl (Me) groups, methoxyethyl (MOE) groups and fluorine (F) groups. These modifications have also been shown to increase resistance to enzyme degradation and improve the stability of DNA–RNA duplex [11, 12, 13]. Lastly, chemical modification of the nucleotide bases, the most common includes modification of the 5′ positions of cytosine, thymine and uracil. Adding methyl groups to cytosine is widely used and is shown to improve binding affinity to the target strand [14].

Bioconjugates have been utilised to link OGNs to biomolecules such as proteins, antibodies and carbohydrates [15, 16, 17]. N‐acetylgalactosamine (GalNAc) conjugates have been shown to greatly improve cellular penetration [14] and aid in targeting the liver and kidneys to treat diseases which impact these organs [18, 19].

OGN therapeutics have great potential to treat a wide range of conditions within the medical landscape. They are complex molecules, and therefore, robust analytical methods must be developed to allow them to reach their potential and ensure their safe use. Impurity profiling of nucleic acid therapeutics is predominantly performed using ion‐pair reverse‐phase high‐performance liquid chromatography (IP‐RP HPLC) with ultra‐violet (UV) detection and mass spectrometry (MS) or tandem mass spectrometry (MS/MS) [20, 21, 22].

IP‐RP HPLC typically utilises tertiary alkylamines ion‐pairing reagents including triethylammonium acetate (TEAA) and tributylammonium acetate (TBuAA) [22, 23, 24, 25]. Using these alkylamines with acetic acid as the counter ion results in high‐resolution separations; however, signal suppression occurs when interfaced with mass spectrometry. Alkylamines with added fluoroalcohols are the most common mobile‐phase component used to improve mass spectrometry sensitivity [24, 26, 27, 28, 29]. Mobile phases using alkylamine and fluoroalcohols such as 1,1,1,3,3,3‐hexafluoro‐2‐propanol (HFIP) as the counter ion results in improved mass spectrometry sensitivity when IP RP separations are interfaced with heated electrospray ionisation mass spectrometry (HESI MS) and offer a powerful approach for characterising and quantifying OGNs therapeutics and their related impurities [26, 30, 31]. The development and application of a wide range of different alkylamine/fluoroalcohol mobile phases have been studied, including the effect of mobile phase ageing [28, 32, 33, 34].

A number of proposed ion‐pair mechanisms and models have been discussed [35, 36, 37, 38, 39]. A dynamic ion exchange model proposed that the IP reagent first adsorbs at the surface of the stationary phase, providing exchange sites for analytes [35, 40, 41, 42]. It was also proposed that both ion‐pairing and dynamic ion exchange occur depending on the experimental conditions [35, 36]. The contribution of ionic interaction in IP‐RP LC depends on the hydrophobicity of IP reagent and is moderated by the amount of organic modifier in the mobile phase. Retention of OGNs in IP RP HPLC was shown to correlate with the alkylamine hydrophobicity [43]. Hydrophobic alkylamines act as more efficient (stronger) IP reagents due to their increased strength of adsorption to the stationary phase compared to less hydrophobic (weaker) IP reagents.

Modulating the mobile phases and IP reagents can be utilised for different purposes. Stronger IP reagents, including tributylamine (TBuA) and hexylamine (HA), are better suited for analysis of phosphorothioate (PS) OGNs as size‐dependent separations largely dominate, reducing the effect of the diastereoisomers present which detrimentally impact chromatographic resolution [24, 25]. Although strong IP alkylamines offer improved chromatography for the analysis of PS OGNs, there are a number of caveats associated with interfacing with mass spectrometry analysis including the formation of ion‐pair adducts, which cause significant polydispersity of the target analyte signal [44]. This reduction of MS signal intensity can lead to difficulty identifying low‐level impurities and small mass changes caused by modification. Strong IP regents, like TBuA [22] and HA, are more prone to adduct formation, which is attributed to their high Henrys law partition coefficient, which results in their low volatility. During droplet formation in the HESI source, the OGN and the alkylamine can desorb as a complex [45, 46]. Removal of these adducts is key to the benefits of strong IP analysis and improved spectra quality via increased signal.

Removing strong ion‐pairs adducts can be challenging; methods for their removal involve optimising ion source settings, such as temperature, gas flow and in‐source collision energy (ce). However, this can also lead to the formation of in‐source impurities in the form of fragmentation ions. Specifically, base loss fragmentation with loss of adenine and guanine commonly seen [47, 48]. Therefore, care is needed to balance the removal of adducts with unwanted fragmentation. Previous work using TBuA utilised different source settings, including drying gas flow rate and temperature, to remove TBuA adducts [22]. These settings were optimised for each sequence individually to achieve the ideal balance of adduct removal and fragmentation.

In this study, we utilised HA:HFIP mobiles phases and optimised HESI source conditions to minimise in‐source generated impurities in the analysis of OGN therapeutics. In‐source conditions were optimised to ensure efficient removal of alkylamine adducts, whilst minimising in‐source fragmentation of OGNs. We have studied the effect of altering a range of HESI parameters including in‐source ce, ion transfer tube (ITT) temperature and vaporiser temperature (VT) on the formation of alkylamine adducts and production of in‐source related impurities in a wide range of different OGNs including ASOs and siRNAs. Furthermore, combined with high‐resolution accurate mass spectrometry (HRAM) analysis and automated deconvolution, a simplified and streamlined approach has been developed for the characterisation of OGN therapeutics and their related impurities.

## 2 Material and Methods

### 2.1 Chemicals and Reagents

Water (UHPLC‐MS grade, Thermo Scientific), acetonitrile (UHPLC‐MS grade, Thermo Scientific), 1,1,1,3,3,3,‐hexafluoro‐2‐propanol (HFIP, >99.8% Fluka LC‐MS grade), hexylamine (HA, 99.7% extrapure Fisher Scientific), tributylamine (TBA, 99%, Acros Organics, Fisher Scientific) and glacial acetic acid (HPLC grade, VWR Chemicals) were used for LC‐MS mobile phases.

### 2.2 Oligonucleotides

OGNs used in this study are shown in Table 1 and were purchased from Eurofins Genomics and MedChemExpress.

**TABLE 1:** Oligonucleotides used in this study. OGN 1 (Patisiran‐antisense siRNA), OGN 2 (Patisiran‐sense siRNA). OGN 3 (Tofersen), OGN 4,5 (fully phoshorothioated 20 mers).

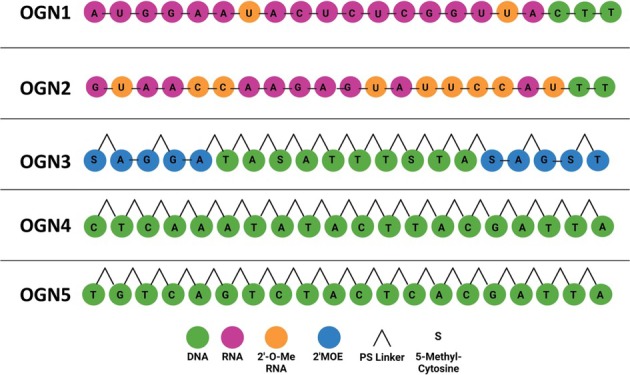

### 2.3 HPLC Analysis

IP RP HPLC analysis was performed on a U3000 HPLC (Thermo Fisher) using a DNAPac RP column (2.1 mm × 100 mm) (Thermo Fisher).

For HA/HFIP mobile phases, mobile phase (A) consisted of 15 mM HA and 60 mM HFIP, and Mobile phase (B) consisted of 15 mM HA, 60 mM HFIP, and 40% ACN,. HPLC separations were performed using a linear gradient starting at 20% (B) and ending at 90% (B) over 14.2 min, flow rate 300 μL/min at 50 °C and UV absorbance 260 nm. Fifty picomoles of OGN 1 and 2 and 100 pmol of OGNs 3, 4 and 5 was injected.

One hundred naomolars tributlyammonium acetate (TBuAA) stock was made using TBA and glacial acetic acid. Mobile phase (A) consisted of 5 mM TBuAA and 10% ACN, and mobile phase (B) consisted of 5 mM TBuAA, and 80% ACN. HPLC separations were performed using a linear gradient starting at 30% B and ending at 65% B over 25 min, flow rate 200 μL/min at 60 °C and UV absorbance 260 nm. One hundred picomoles of OGN 3 injected.

### 2.4 Mass Spectrometry Analysis

Mass spectrometry analysis was performed using an Orbitrap Exploris 480 (Thermo Fisher) or Orbitrap Exploris 240 (Thermo Fisher Scientific). The MS settings include a vaporiser temperature of 300 °C, sheath gas set to 35 arbitrary units, aux gas set to 10 arbitrary units and sweep gas set to 5 arbitrary units unless otherwise stated. MS detection was performed in both negative and positive mode. The scan range was 400–2500 m/z. The ITT temperature was set to 275 °C, and the VT was set to 300 °C as default and varied accordingly. In‐source ce was varied between 0 and 65 V for HA/HFIP in negative mode, 30 and 90 V for HA/HFIP in positive mode and 0–65 V for TBuAA in negative mode. Data was acquired in the peptide application mode at standard pressure (1 arbitrary unit), and the resolution was set to 120 000. The RF lens percentage was set to 70.

### 2.5 Data Analysis

The Intact Protein Deconvolution function in Chromeleon 7.3.1 or 7.3.2 was used for deconvolution using XTRACT sliding windows deconvolution with the following settings: rel. intensity threshold = 1%, target avg. spectrum width = 0.390 min, number of detected intervals = 3, merge tolerance = 30 ppm, max RT gap = 1, output mass range = 1000–8000, S/N threshold = 3, charge range = 2–15 (OGN1 + 2), 2–10 (OGNs 3, 4 and 5), min number detected charge = 2 and rel abundance threshold = 0. For each OGN, the RT widow and m/z range were set individually to match the LC‐MS method. Custom columns, target mass (Da) and target tolerance (Da) were added to the sequence manager window. Target mass was set to the mass of the sample, and target tolerance was set to 1. A custom impurities table featuring the ions specific to this analysis was used with the reporting feature to quantify the target mass, IP adducts and fragmentation ions.

## 3 Results and Discussion

### 3.1 Optimisation of LC HESI MS Source Conditions for the Analysis of OGN Therapeutics in Negative Mode Ionisation

**FIGURE 1:**
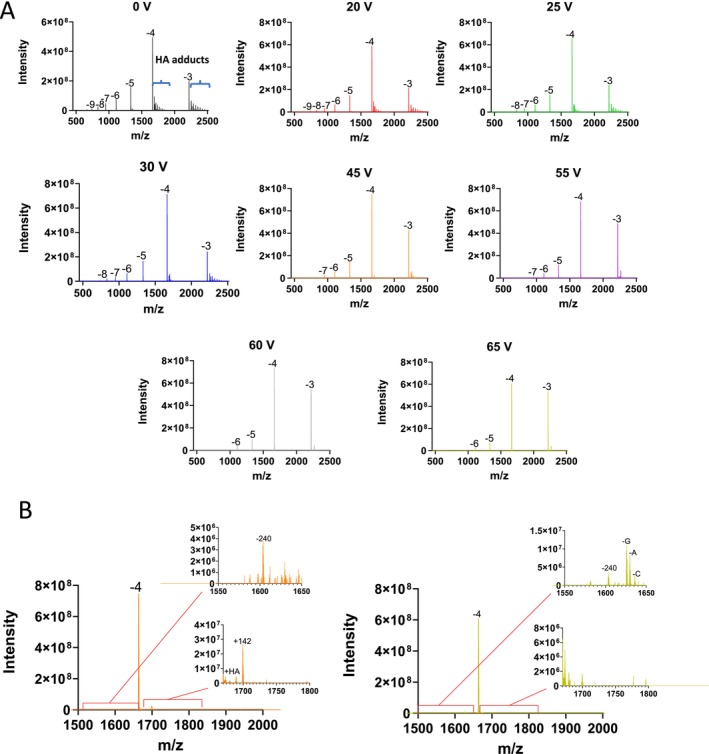
Effects of in‐source energy on alkylamine adducts and in‐source fragmentation of OGN1 (Patisiran‐antisense siRNA). (A) HESI MS spectra at a range of in‐source ce (0, 20, 25, 30, 45, 55, 60 and 65 V). (B) HESI MS spectra of the −4 charge state of OGN1 at 45 V and 60 V. Inset panels highlight the alkylamine adducts and corresponding OGN fragmentation and base loss.

A range of different OGNs including therapeutic OGNs were analysed using IP‐RP LC‐MS in conjunction with 15 mM HA/60 mM HFIP mobile phases. Under these conditions, size‐dependent separations largely dominate, reducing the effect of the diastereoisomers present in phosphorothioate (PS) OGNs, which detrimentally impact chromatographic resolution of the full length product from associated manufacturing impurities. Initial work focused on the effect of in‐source ce setting on an Orbitrap Exploris mass spectrometer on HA adduct removal and in‐source fragmentation of the corresponding OGN in negative ion mode. The LC‐MS spectra obtained for OGN1 (Patisiran‐antisense siRNA a 21 mer ssRNA partially 2’‐O‐methylated, see Table 1) over a range of different in‐source ce (0–65 V) is shown in Figure 1A,B. The results show that under low or no in‐source ce the presence of HA adducts are observed and are predominantly on the lowest charge states present, while no significant HFIP or metal ion adducts were observed. This results in polydispersity of the signal on the low charge states. A combination of reduced coulombic repulsion, favourable conformational states, and solvation effects in lower charge states of the OGN ions makes the formation of alkylamine adducts more likely in these charge states during negative ion HESI. At 0 V, the presence of one to three HA adducts are highlighted (see Figure 1A, top left panel). As the in‐source ce is increased, a reduction of HA adducts is observed. However, as in‐source ce increases, this causes an increase OGN fragmentation with a predominant loss of adenosine (−A) and guanine (−G) consistent with the known gas phase fragmentation behaviour of OGNs [48]. Nucleobases can be lost as neutral or negatively charged species in the gas phase. Previous work has shown that the preference for neutral or charged base loss depends on a number of factors, including the charge state of the OGN, sequence position and whether the OGN is DNA or RNA [49, 50, 51]. For the analysis of OGN1 (siRNA), m/z values corresponding to the [M‐ 5H‐G^−^]^4−^ were observed for charged base loss of G, neutral bass loss was observed for A and C with m/z values corresponding to [M‐4H‐B(A)]^4−^ and [M‐4H‐B(C)] [4, 5, 6, 7, 8, 9, 10, 11, 12, 13, 14, 15, 16, 17, 18, 19, 20, 21, 22, 23, 24, 25, 26, 27, 28, 29, 30, 31, 32, 33, 34, 35, 36, 37, 38, 39, 40, 41, 42, 43, 44, 45, 46, 47, 48, 49] (see Figure 1B and Supplementary Figure S1A). In addition, as ce is increased, a reduction in signal intensity of the higher charge states (−8, −9) is observed, consistent with the reduced stability of the higher OGN charge states [52, 53]. The greater coulombic repulsion of the higher charge states leads to significantly lower values for the critical energy of fragmentation.

LC‐MS spectra are shown for representative in‐source ce settings of 45 V and 65 V for the corresponding 4− charge state (see Figure 1B). The results show the under optimised ce settings of 45 V, this results in the effective reduction of HA adducts and no significant in‐source OGN fragmentation (base loss) is observed. However, at higher ce voltages, increased OGN fragmentation (base loss) is observed, therefore generating significant in‐source impurities.

### 3.2 Effect of HESI and Source Temperature on Alkylamine Adduct Removal in Conjunction With In‐Source Collision Energy

The results above demonstrated that using HA/HFIP mobile phase with optimised in‐source ce voltages minimised LC‐MS generated impurities (HA adducts and OGN fragmentation). Therefore, additional experiments were performed to study the effects of additional HESI parameters including ion transfer tube (ITT) temperature and vaporiser temperatures (VT) in conjunction with in‐source ce on the Orbitrap Exploris mass spectrometer.

**FIGURE 2:**
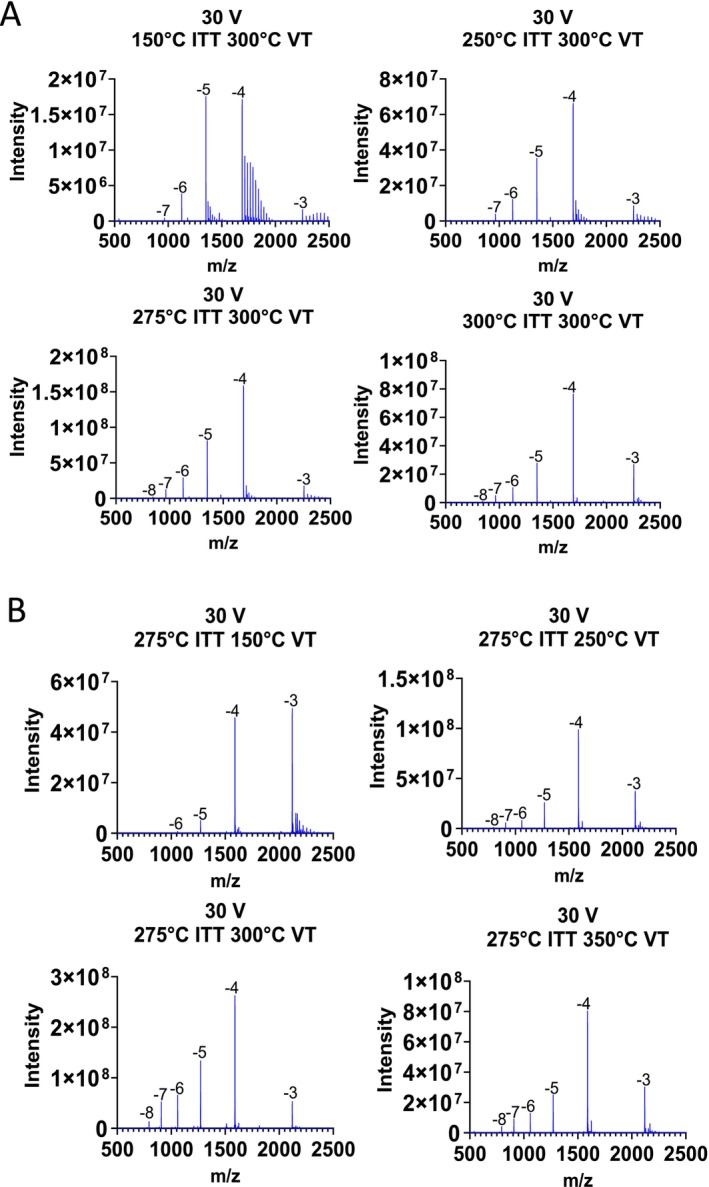
Effects of ITT temperature and VT on alkylamine adducts and in‐source fragmentation. (A) HESI MS spectra of OGN2 (Patisiran‐sense siRNA) over a range of ITT temperatures (150 °C, 250 °C, 275 °C and 350 °C) at constant VT of 300 °C and ce set at 30 V. (B) HESI MS spectra of OGN2 (Patisiran‐sense siRNA) over a range of VT (150 °C, 250 °C, 300 °C and 350 °C) at constant ITT temperature of 275 °C and ce set at 30 V.

A range of different ITT temperatures (150 °C, 250 °C, 275 °C and 350 °C) were studied at a constant VT of 300 °C and ce set at 30 V (see Figure 2A). The results show that for the analysis of OGN2 (Patisiran‐sense siRNA) as ITT temperature is increased, alkylamine adducts present on the lower charge states (3−, 4−) decreased and no significant OGN fragmentation was observed across all ITT temperatures. The effect of VT was also investigated, ITT temperature was kept constant at 275 °C and in‐source ce at 30 V and the VT was varied at 150 °C, 250 °C, 300 °C and 350 °C (see Figure 2B). The results show that similar to previous alterations in ITT temperature, as the VT increases, the abundance of alkylamine adducts present on the lower charge states (3−, 4−) decreases and no significant OGN fragmentation was observed across all vaporisation temperatures used shown in Figure 2B. The results show that at lower vaporisation temperature (150 °C) significantly higher alkylamine adducts were present compared to the lowest ITT temperature (150 °C) used. The VT also impacts the observed charge state distribution of the OGN, the results show that the −8, −7 charge states were only observed above 300 °C for OGN2 under the conditions analysed (see Figure 4B). These results were consistent with the analysis of OGN4 across varying ITT temperatures and vaporiser temperatures (see Supplementary Figure S3A/B).

The results show that at higher ITT and vaporiser temperatures, less alkylamine adducts are observed on the lower charge states, consistent with a reduction in alkylamine binding to the phosphodiester backbone of the OGN. The alkylamine‐OGN complexes are not amenable for further deprotonation by counter ion in the gas phase, leading to lower charge states. Increasing source temperature reduces the alkylamine‐OGN complexes enabling further deprotonation of the free OGN by counterion in the gas phase, leading to high charge states [46]. Alternative proposed mechanisms can also explain the effects observed as linear alkylamines (such as HA) are proposed to keep the OGNs in a more folded conformation due to neutralisation of the phosphate backbone [46]. This, therefore, results in less charge repulsion; HA therefore enhances ionisation via the charge residual model (CRM) as opposed to the chain ejection model (CEM) [54], which results in lower charge states. This proposed mechanism is consistent with data where at lower vaporiser/ITT temperatures, increased amounts of HA are bound to the OGN resulting in neutralisation of the phosphate backbone enhancing ionisation via the CRM resulting in lower charge states. At higher ITT and VT (more denaturing conditions), reduced amounts of HA are bound to the OGN generating more highly charged OGNs that unfold and promote ionisation via the CEM resulting in higher charge states.

However, this is in contrast to previous data where an increase in higher charge states using octylamine:nonafluoro‐tert‐butyl alcohol was observed at lower source temperature conditions, 80 °C and 100 °C compared 120 °C [55]. This supports the idea that these HESI droplet microenvironments can be significantly affected by the mobile phase and source conditions, resulting in changes to the observed HESI mass spectra [56].

Following high‐resolution mass spectrometry analysis of the OGN and optimisation of the in‐source settings, a rapid streamlined mass deconvolution and data analysis workflow was developed in Chromeleon to identify and quantify impurities present based on HRAMS analysis (see Supplementary Figure S2).

### 3.3 Deconvolution Parameters for Identification and Quantification of OGN Impurity Masses

From the data and optimisation described above, in‐source settings can influence the charge state distribution, alkylamine adduct removal and in‐source impurity generation. The ultimate goal would be to remove all the alkylamine adducts while preventing any generation of in‐source impurities. This is dependent on the OGN sequence and structure and usually cannot be achieved for the lower charge states. The higher charge states on the other hand, either do not have any adducts to begin with or they are removed much more easily. Using optimised source conditions and higher eluent pH values to generate the higher charge states, enables the utilisation of these higher charges states for deconvolution whilst ignoring the lower charge states that carry the adducts. There are two parameters in the deconvolution settings which can be exploited to focus the deconvolution on ignoring potential adducts in the final results. This allows milder source conditions to be employed which prevent in‐source generated impurities but may still leave some adducts present on the lower charge states.

Having multiple higher charge states available for deconvolution prevents the necessity of requiring the lowest charge state for deconvolution. The first parameter that can be employed is the m/z range, this can be set so that the higher value falls below the m/z of the lowest charge state. This would effectively put the lowest charge state out of the m/z range of the deconvolution. The second setting is the minimum number of detected charges. This specifies the minimum number of charge states required to produce a deconvolution result. No deconvolution results present with less than this minimum number of charge states appear in the results list. Which means if this is set to a value of 2, if a single low charge state is the only charge state that contains adducts, the non‐adduct m/z for this charge state would be used in the deconvolution but the m/z values from the adducts would not appear in the deconvoluted results as they are only present under one charge state.

Using these parameters for deconvolution allows mild source settings which may still leave adduct formation on some low charge states, but there would be no in‐source generation of impurities. This is important as source generation of abasic impurities would leave doubt about the impurity being present in the original sample or not. Figure 1A shows the full charge state spectrum of OGN1 analysed with an in‐source ce of 45 V. The alkylamine adducts are clearly visible on the low charge state [−4 and −3] but are absent in all the other charge states. Using the above‐described parameters in the deconvolution settings, the deconvoluted results show all the impurities expected from the sample without any adducts or in‐source generated impurities. With optimisation of the source conditions to focus on prevention of in‐source impurity generation, whilst removing as many adducts as possible, the software parameters can deal with any remaining amine adducts.

**FIGURE 3:**
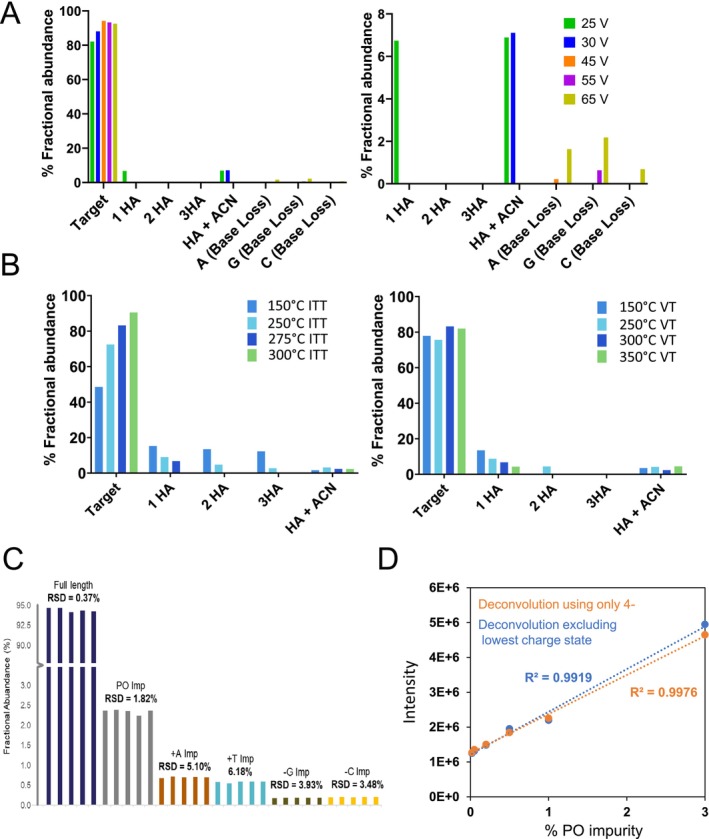
Deconvolution parameters for the quantification of OGNs and their related impurities. (A) % Fractional abundance of the alkyamine adducts and gas‐phase base loss are shown for varying in‐source voltages following deconvolution of charge states −4 and above. (B) % Fractional abundance of an OGN and gas‐phase base loss are shown for a range of ITT temperatures (left) and a range of VT (right), following deconvolution of charge states −4 and above. (C) % fractional abundance of OGN impurities. Five replicate injections of a phosphorothioated OGN are shown and deconvolution was performed using charge states −4 and above. (D) Comparative analysis of the intensity of the PO impurity (0–3%) spiked into a phosphorothioated OGN. Deconvolution was performed using charge states −4 and above (shown in orange) and only the most abundant charge state (−4 shown in blue).

Following mass deconvolution as described above a summary of the quantification of the HA adducts and OGN base loss over a range of source ce for OGN1 is shown in Figure 3A. Consistent with LC‐MS spectra previously observed, the results show that alkylamine adducts are only present at low ce voltages. As ce is increased, there is an increase in the % abundance of in‐source generated impurities where gas‐phase base loss of A and G are the most abundant. Under these conditions, an optimum ce of 45 V is determined for effective alkylamine adduct removal and with no significant OGN fragmentation (base loss observed) resulting in the highest % fractional abundance of the OGN target mass and therefore minimum in‐source generated impurities.

In addition, a summary of the quantification of the HA adducts and OGN base loss over the range of different source conditions following mass deconvolution is shown in Figure 3B and Supplementary Figure S3C. The results highlight the decrease in alkyamine adducts at higher ITT and VTs as described above.

The ability to detect low level impurities in an OGN sample using the streamlined deconvolution settings is demonstrated in Figure 3C. The results show a series of replicate 1 μg injections of a phophorothioated OGN and the fractional abundance of the FLP and low level impurities including the PO. Further analysis using the deconvolution parameters excluding the lowest charge state was compared to deconvolution using only the most abundant charge state (4−) for the quantification of low level PO impurities. OGN samples were generated using a phosphorothioated OGN with the addition of a PO impurity spiked in (0.02 to 3%). Quantification of the PO impurity was determined using both of the deconvolution approaches described above (see Figure 3D). The results show consistent linearity and similar relative intensities determined for the PO impurities across the different deconvolution settings.

### 3.4 Optimisation of LC HESI MS Source Conditions for the Analysis of ASO OGNs

**FIGURE 4:**
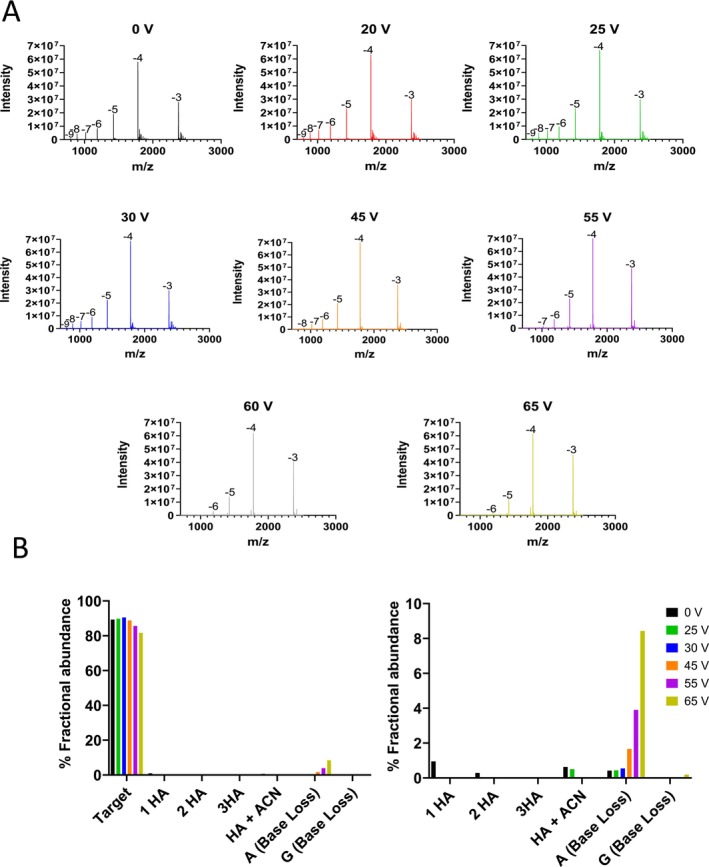
Effects of in‐source energy on alkylamine adducts and in‐source fragmentation of OGN3 (Tofersen). (A) HESI MS spectra at a range of in‐source ce (0, 20, 25, 30, 45, 55, 60 and 65 V). (B) % Fractional abundance of the alkyamine adducts and base loss are shown for varying in‐source voltages following deconvolution of charge states −4 and above.

Further LC‐MS analysis was performed using an alternative OGN therapeutic (Tofersen‐a 20 nt OGN with 2’ OMe, 2’ MOE and PS modifications) see Table 1 OGN3. The OGN was analysed using LC‐MS under a range of different in‐source collision energies as previously described (see Figure 4A,B). The results show that under these conditions, the predominant charge state observed was 4−. The results show the presence of HA adducts on the low charge states at low ce settings when no in‐source ce is applied (see Figure 4A). The HA adducts are effectively removed as the in‐source ce increases with a reduction in signal intensity of the higher charge states (9− to −7) consistent with previous observations. Following mass deconvolution as previously described, a summary of the quantification of the HA adducts and OGN base loss over a range of source ce is shown in Figure 4B. Under these conditions for the analysis of Tofersen, the optimum ce where a reduction of HA adducts and minimising OGN fragmentation (generation of in‐source impurities) was obtained at 30 V as highlighted by the highest fractional abundance of the target mass of the OGN FLP (see Figure 2B). At high collision energies (>30 V) m/z values corresponding to the [M‐ 4H‐B(A)]^4−^ was the predominant species for this OGN (see Figure 2B and Supplementary Figure S1B). The abundant neutral base loss (A) observed for this OGN at high in‐source ce is in contrast to the most abundant charged base loss of G which was observed for the siRNA at high in‐source ce.

Finally, LC‐MS analysis of a fully phosphorothioated OGN (OGN4) was studied over a range of in‐source ce settings (see Supplementary Figure S4A/B). As previously observed as in‐source ce is increased, a decrease in HA adducts is observed which are present predominately on the lower charge states. At high ce voltages (60 V), a clear reduction or removal of the adducts is observed in conjunction with a significant change in charge series compared to low or 0 V. Significant base loss was observed at high ce, as an m/z value corresponding to the [M‐4H‐B(A)]^4−^ was observed, demonstrating neutral base loss (predominantly A) (see Supplementary Figure S1C and 4B).

In summary, the results show that analysis of a range of OGNs using HA/HFIP mobile phases in conjunction with optimised ce voltages and streamlined accurate mass deconvolution enables the identification and quantification of OGNs and their related impurities. Under optimised (mild) in‐source ce settings enables a reduction in the LC‐MS generated impurities via the presence of HA adducts from the mobile phase and in‐source generated impurities from the OGN fragmentation (gas phase base loss). Optimisation of the appropriate ce settings is dependent on the sequence and chemical composition of the OGN. The LC‐MS data indicate that the generation of in‐source impurities is dependent on the stability of the OGN in the gas phase. The results shows the enhanced stability of Patisiran ‐antisense siRNA and Tofersen compared to fully phosphorothioated ASO (ssDNA). It has been shown previously that base loss is less prevalent in RNA due to the 2′‐hydroxyl on the ribose stabilising the N‐glycosidic bond [57]. Furthermore, proposed mechanisms for base loss, such as neutral loss involves the 2′ hydrogen [58]. Therefore, the increased gas phase stability for Patisiran‐antisense siRNA and Tofersen compared to fully phosphorothioated ASO can be attributed to the 2′ modifications of the ribose sugars, stabilising the glycosidic bond and preventing subsequent base loss. These findings are consistent with previous studies in which MOE modifications improve stability under harsher conditions in terms of drying gas flow rate and temperature [22].

### 3.5 Comparative LC MS Analysis of OGNs Using HA/HFIP and TBuAA Mobile Phases

**FIGURE 5:**
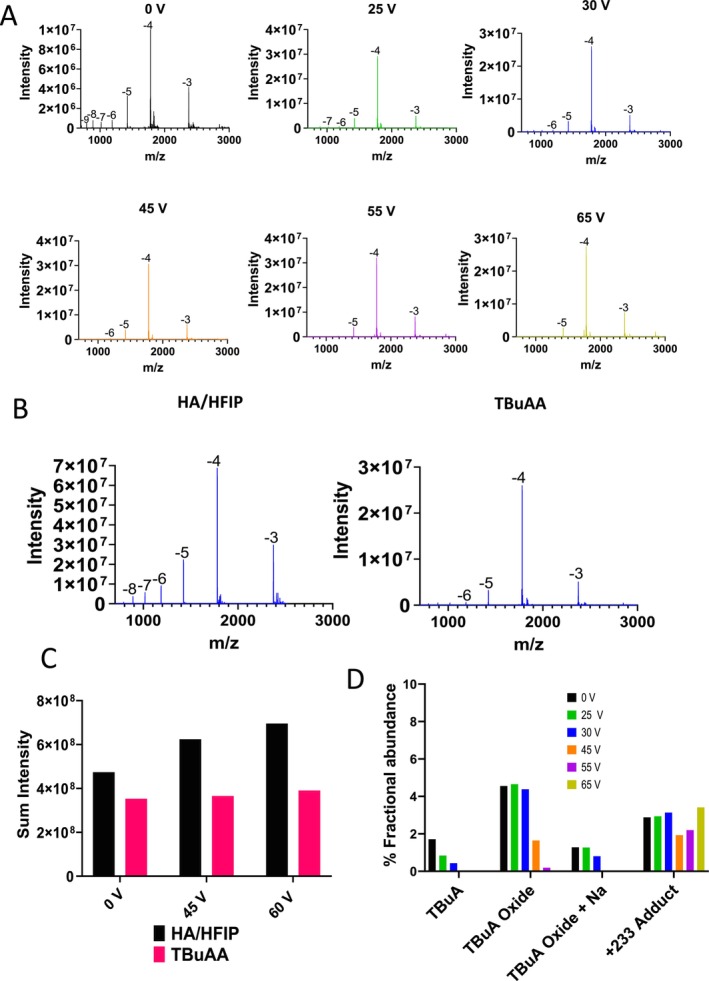
Comparative LC‐MS analysis of OGN3 (Tofersen) using TBuAA and HA/HFIP. (A) HESI MS spectra at a range of in‐source ce (0, 25, 30, 45, 55 and 65 V) using TBuAA. (B) HESI MS spectra using in‐source ce 30 V for HA/HFIP (left) and TBuAA (right). (C) Comparative sum intensity of the HESI MS spectra of OGN3 (Tofersen). One hundred picomoles of OGN2 was analysed and normalised against UV peak area. (D) % Fractional abundance of the alkyamine adducts are shown for varying in‐source voltages following deconvolution of charge states −4 and above in TBuAA.

Following optimisation of the HESI source settings and streamlined deconvolution methods for impurity profiling, a comparison was performed against the commonly used TBuAA mobile phase [22]. OGN3 was analysed using 5 mM TBuAA and compared against the HA/HFIP (see Figure 5). For the analysis using TBuAA (neutral pH) across a range of different in‐source ce, the expected shift to the lower charge states is observed (see Figure 5A). The comparative data of the same source settings for the analysis of the OGN in TBuAA and HA/HFIP is shown in Figure 5B. It is interesting to note that the abundance of the 5− charge state in HA/HFIP is of similar intensity to the most abundant 4− charge state observed in 5 mM TBuAA. Figure 5C shows a comparison of the total signal intensity and shows that there is an increase in signal intensity achieved using the HA/HFIP mobile phases in comparison to the TBuAA. The results also demonstrate the increased abundance of the higher charge states using HA/HFIP, where alkylamine and metal ion adducts are less prevalent.

The data also shows that in the absence or low in‐source ce, differences are observed in the abundance of alkylamine adducts. Focussing on the 4− charge state, increased abundance (and multiple) HA adducts are observed in comparison to the TBA and TBA oxide adducts observed. The increased abundance of the HA adducts likely reflects the higher concentration of HA used compared to TBA (15 mM vs 5 mM respectively). Therefore, these results also demonstrate the importance of optimising HESI parameters as outlined previously using the HA/HFIP mobile phases to effectively remove the HA adducts whilst minimising in‐source fragmentation and taking advantage of the benefits of the higher charge states observed and increased signal intensity (ionisation efficiency). Finally, the comparison also shows that as ce is increased, the HA adducts are effectively removed from the −4 charge state at 45 V (see comparison with 0 V data) and are not present on the higher charge states across the majority of ce used. In contrast, the TBA and TBA oxide adducts are still present on the −4 charge state at 45 V and above (see Figure 5D and Supplementary Figure S6A‐C).

### 3.6 Effect of In‐Source Collision Energy on Adduct Levels in Positive Mode Ionisation

**FIGURE 6:**
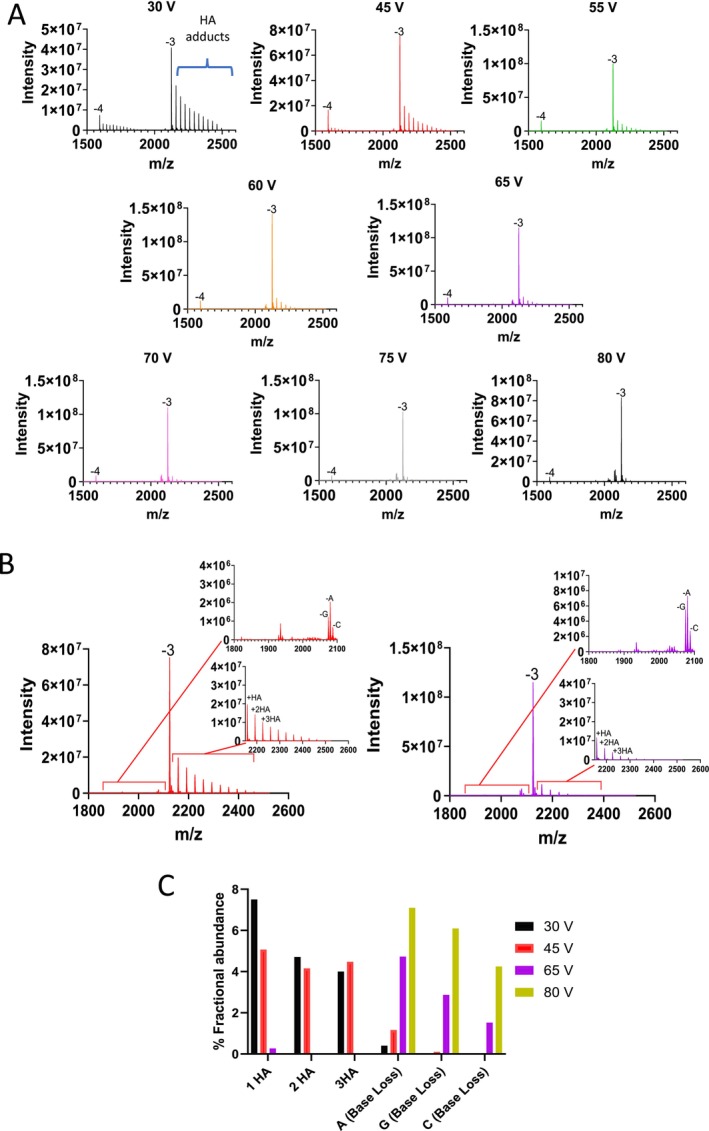
LC‐MS analysis in positive ion mode. (A) HESI MS spectra of OGN4 (21 mer ssDNA phosphorothioate) over a range of in‐source ce (30–80 V). (B) HESI MS spectra of the −3 charge state of OGN3 at 45 and 65 V respectively. Inset panels highlight the alkylamine adducts and corresponding OGN fragmentation and base loss. (C) % Fractional abundance of the alkyamine adducts and gas‐phase fragmentation (base loss) in positive ionisation mode. Varying in‐source ce (30 V, 45 V, 65 V and 80 V) were used for the analysis of OGN 3 (ssDNA phosphorothioate) prior to deconvolution and data analysis to determine the % fractional abundance of the alkyamine adducts and gas‐phase fragmentation (base loss).

Negative mode ionisation HESI is predominantly used for analysing OGNs owing to their polyanionic nature, resulting in improved ionisation efficiency and greater signal intensity. However, positive mode HESI has been demonstrated for the analysis of OGNs [59, 60]. Applications of positive mode HESI MS include the analysis of Morpholino antisense OGNs (absence of negatively charged phosphodiester backbone) which favours positive ion formation [61]. In this study, we also used positive ion mode LC HESI MS to analyse the OGN therapeutics under the same mobile phase conditions as described above (15 mM HA, 60 mM HFIP) and analysed the effect of in‐source collision energies on alkylamine adducts and in‐source fragmentation of the OGN. Figure 6 and Supplementary Figure S7 show the analysis of OGN1, 4 and 5 analysed in positive mode at in‐source collision energies of 30–80 V. The results for the analysis of OGN4 demonstrate that in positive mode, additional alkylamine adducts are present on the limited lower charge states observed compared to negative mode, generating significant polydispersity of the OGN signal. Higher in‐source collision energies are required to remove the alkylamine adducts. The results show that as ce was increased, the amount of adducts present decreased, following the trend demonstrated in negative mode.

Similar to previous observations in negative ion mode, the main form of fragmentation is base loss, with the purines again the most labile, neutral base loss (A) was the most abundant base loss observed for this OGN. However, greater stability is seen in positive mode, and therefore, higher in‐source ce voltages can be applied before similar levels of base loss in negative mode are seen. This is clearly demonstrated with both the qualitative and quantitative analysis, where significant base loss only beginning to be seen at 65 V in positive mode. In addition, at 45 V, which was the optimum condition in negative mode ionisation for OGN4 (see Supplementary Figure S4), in positive mode, a significant amount of adducts are still present at this voltage. For positive mode, the optimum conditions were between 65 and 70 V, which saw the majority of alkylamine adducts removed and minimising the generation of in‐source impurities (see Figure 6). In negative mode, high voltages such as this would cause significant in‐source impurities. It is interesting to note that under optimised ce conditions for both positive and negative HESI MS, there is no significant difference in signal intensity for the same amount for OGN analysed.

## 4 Conclusions

OGN therapeutics are an emerging class of drugs that have the potential to treat a wide range of diseases. Characterisation of OGN therapeutics and their related impurities is predominantly performed using IP‐RP HPLC interfaced with mass spectrometry. In this study, we have developed a sensitive LC‐MS method for analysis of antisense OGNs and small interfering RNAs utilising HA/HFIP mobile phases in conjunction with optimised mild HESI source conditions to minimise in‐source generated impurities and alkylamine adducts in the analysis of OGN therapeutics.

Source conditions were optimised to ensure efficient removal of alkylamine adducts whilst minimising in‐source fragmentation of OGNs. We have studied the effect of altering a range of HESI parameters on the formation of alkylamine adducts and the production of source‐related impurities in a wide range of different OGNs, including ASOs and single stranded siRNAs. Optimisation of the appropriate source settings was shown to be dependent on the sequence and chemical composition of the OGN. The results show that under low or no in‐source ce the presence of HA adducts are observed and are predominantly on the lowest charge states present. As the in‐source ce is increased, a reduction of HA adducts is observed. However, as in‐source ce increases, this causes an increase in nucleobase loss in the gas phase, therefore generating in‐source impurities.

Utilising HA/HFIP mobile phase results in increased overall HESI MS sensitivity compared to TBuAA mobile phase under the conditions used in this study. Using optimised source conditions in conjunction with the higher eluent pH of HA/HFIP mobile phases, results in higher charge states, which can be utilised for deconvolution whilst ignoring the lower charge states that carry the adducts.

Further analysis of the OGNs using HA/HFIP mobile phases in positive ion mode showed that additional alkylamine adducts are present on the limited lower charge states observed compared to negative mode, generating significant polydispersity of the OGN signal. Higher in‐source collision energies are required to remove the alkylamine adducts compared to those optimised in negative ion mode for the same corresponding OGN. However, greater stability is seen in the positive mode, and therefore, higher in‐source ce voltages can be applied before similar levels of base loss in the negative mode are seen. Comparing positive and negative HESI MS, there was no significant difference in signal intensity under optimised conditions for the same amount of OGN analysed.

Optimisation of the mild source conditions in conjunction with high pH mobile phases was combined with high‐resolution accurate mass spectrometry analysis and automated deconvolution workflow. This simplified and streamlined approach for characterising OGN therapeutics and their related impurities, without any significant adducts present in the deconvoluted provides a rapid universal method for impurity analysis with automated GLP compliant data handling.

## Author Contributions

**Mollie A. Glenister:** investigation, writing – original draft, writing – review and editing, methodology. **Ulrik Mistarz:** investigation, software, writing – review and editing. **Ken Cook:** investigation, supervision, writing – review and editing, conceptualization. **James L. Stephenson:** writing – review and editing, supervision. **Mark J. Dickman:** conceptualization, funding acquisition, writing – original draft, writing – review and editing, supervision.

### Peer Review

The peer review history for this article is available at https://www.webofscience.com/api/gateway/wos/peer‐review/10.1002/rcm.10033.

## Supporting information


**Data S1** Supporting Information

## Data Availability

The data that support the findings of this study are available from the corresponding author upon reasonable request.
