# Supplementary material for: Optimisation of Heated Electrospray Ionisation Parameters to Minimise In‐Source Generated Impurities in the Analysis of Oligonucleotide Therapeutics
Source: Rapid Commun Mass Spectrom. 2025 Apr 3;39(13):e10033. doi: 10.1002/rcm.10033 (PMC11969060; doi:10.1002/rcm.10033)
Supplement: Supplementary file 1 — Data S1 Supporting Information [file RCM-39-e10033-s001.pptx]

## Slide 1
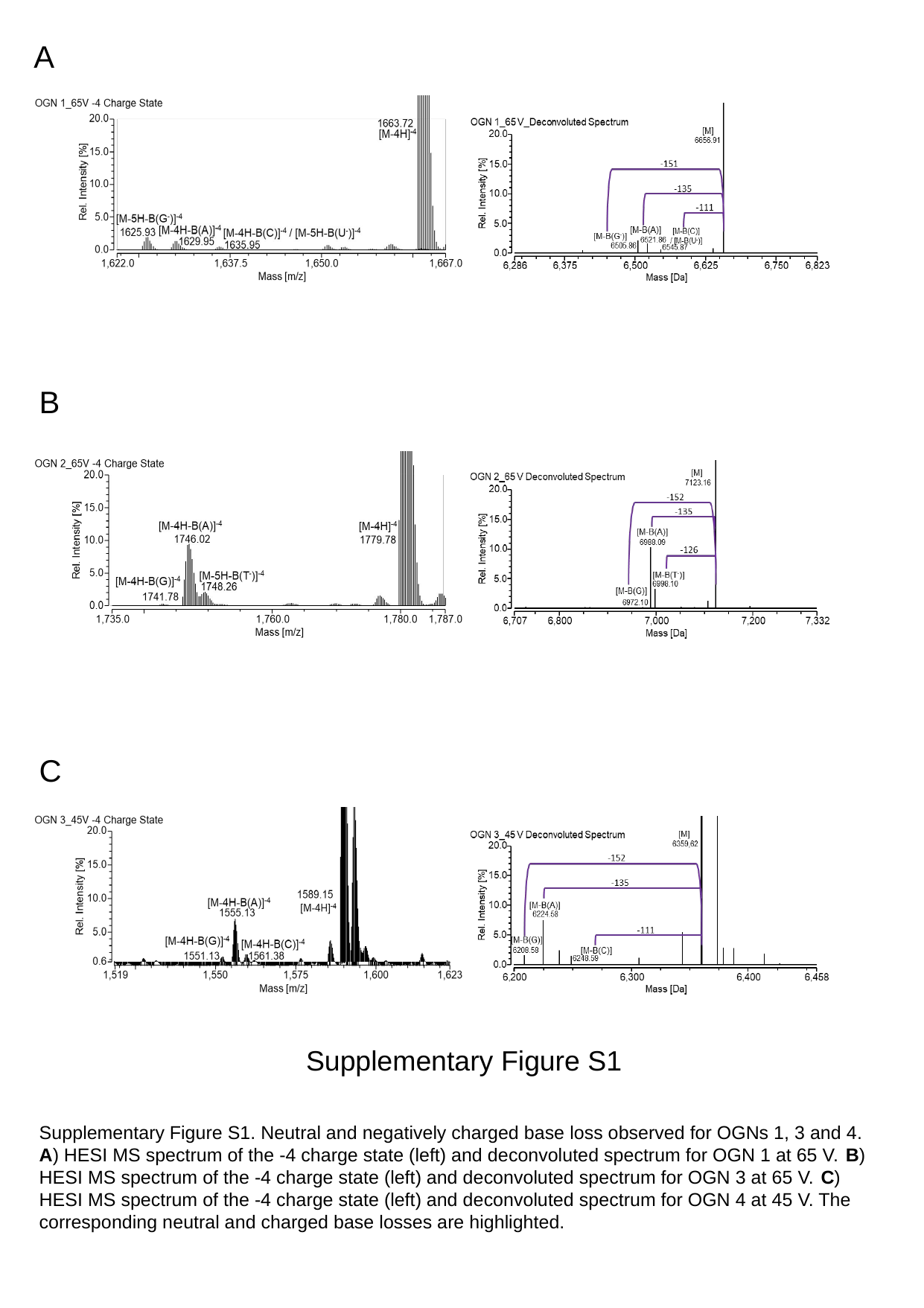

A
B
C
Supplementary Figure S1
Supplementary Figure S1. Neutral and negatively charged base loss observed for OGNs 1, 3 and 4. A) HESI MS spectrum of the -4 charge state (left) and deconvoluted spectrum for OGN 1 at 65 V. B) HESI MS spectrum of the -4 charge state (left) and deconvoluted spectrum for OGN 3 at 65 V. C) HESI MS spectrum of the -4 charge state (left) and deconvoluted spectrum for OGN 4 at 45 V. The corresponding neutral and charged base losses are highlighted.

## Slide 2
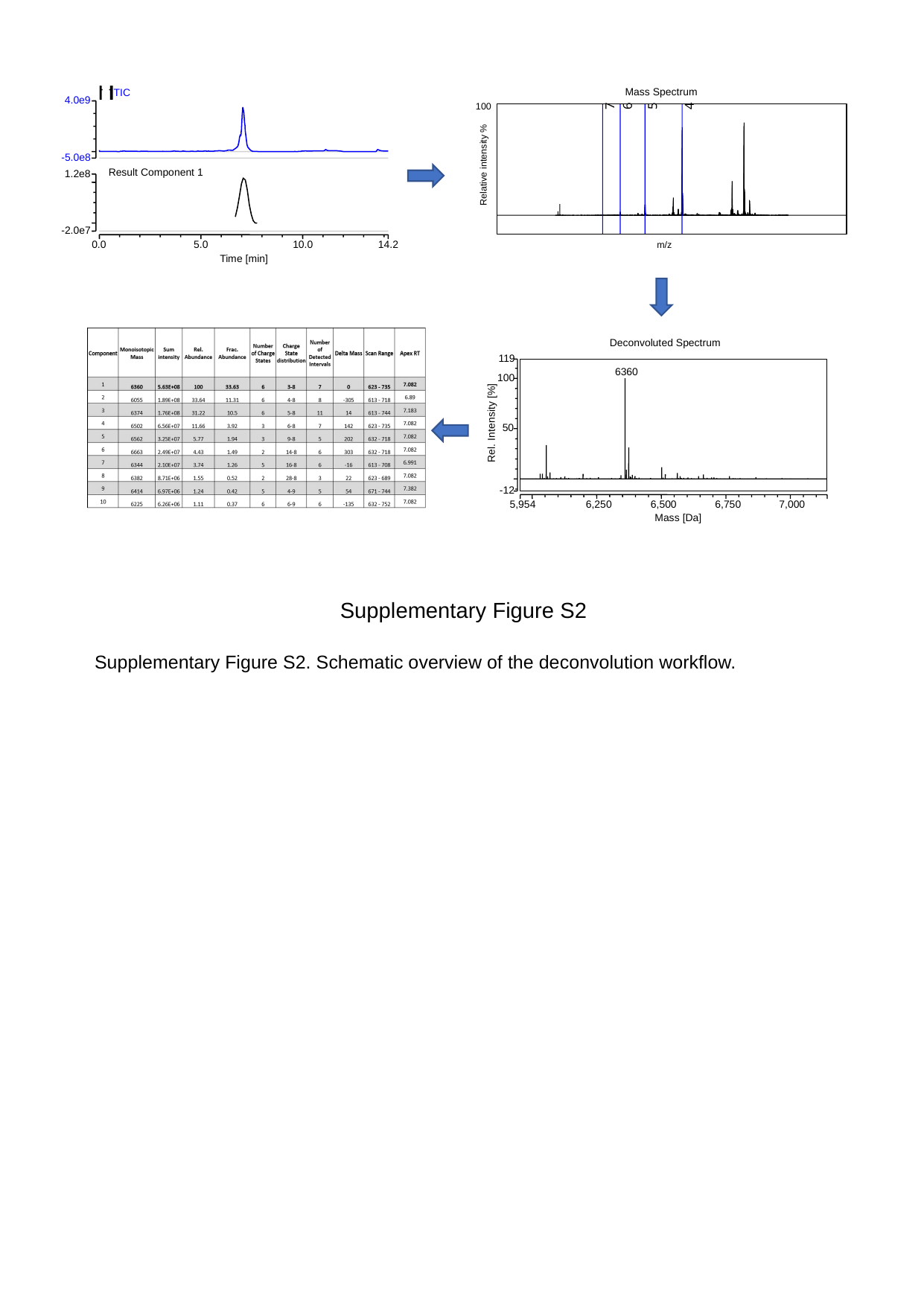

Mass Spectrum
TIC
4.0e9
-5.0e8
Result Component 1
1.2e8
-2.0e7
0.0
5.0
10.0
14.2
Time [min]
100
7
6
5
4
Relative intensity %
m/z
Deconvoluted Spectrum
119
100
]
%
[
y
t
i
s
n
e
50
t
n
I
.
l
e
R
-12
5,954
6,250
6,500
6,750
7,000
Mass [Da]
6360
Supplementary Figure S2
Supplementary Figure S2. Schematic overview of the deconvolution workflow.

## Slide 3
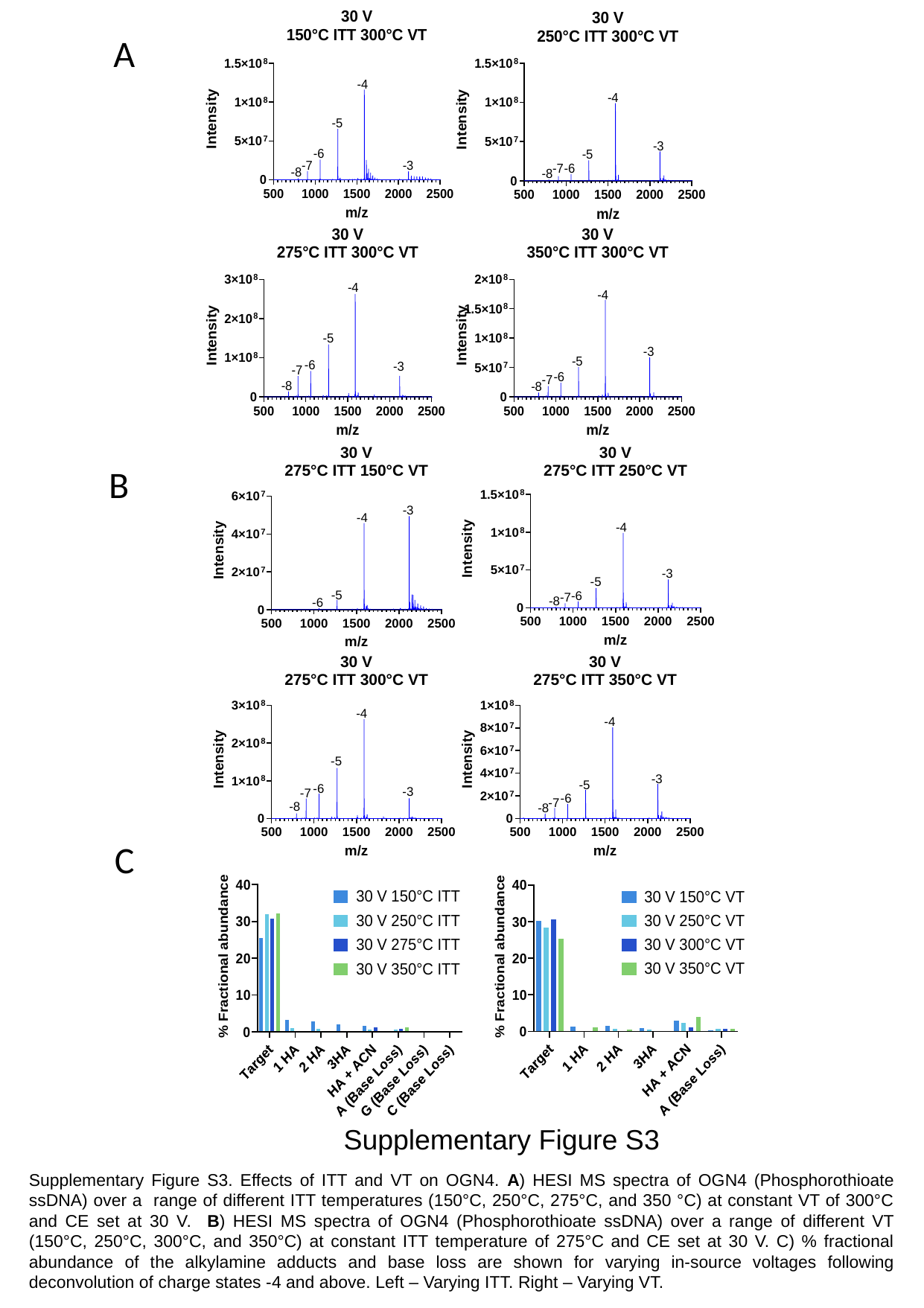

A
B
C
Supplementary Figure S3
Supplementary Figure S3. Effects of ITT and VT on OGN4. A) HESI MS spectra of OGN4 (Phosphorothioate ssDNA) over a range of different ITT temperatures (150°C, 250°C, 275°C, and 350 °C) at constant VT of 300°C and CE set at 30 V. B) HESI MS spectra of OGN4 (Phosphorothioate ssDNA) over a range of different VT (150°C, 250°C, 300°C, and 350°C) at constant ITT temperature of 275°C and CE set at 30 V. C) % fractional abundance of the alkylamine adducts and base loss are shown for varying in-source voltages following deconvolution of charge states -4 and above. Left – Varying ITT. Right – Varying VT.

## Slide 4
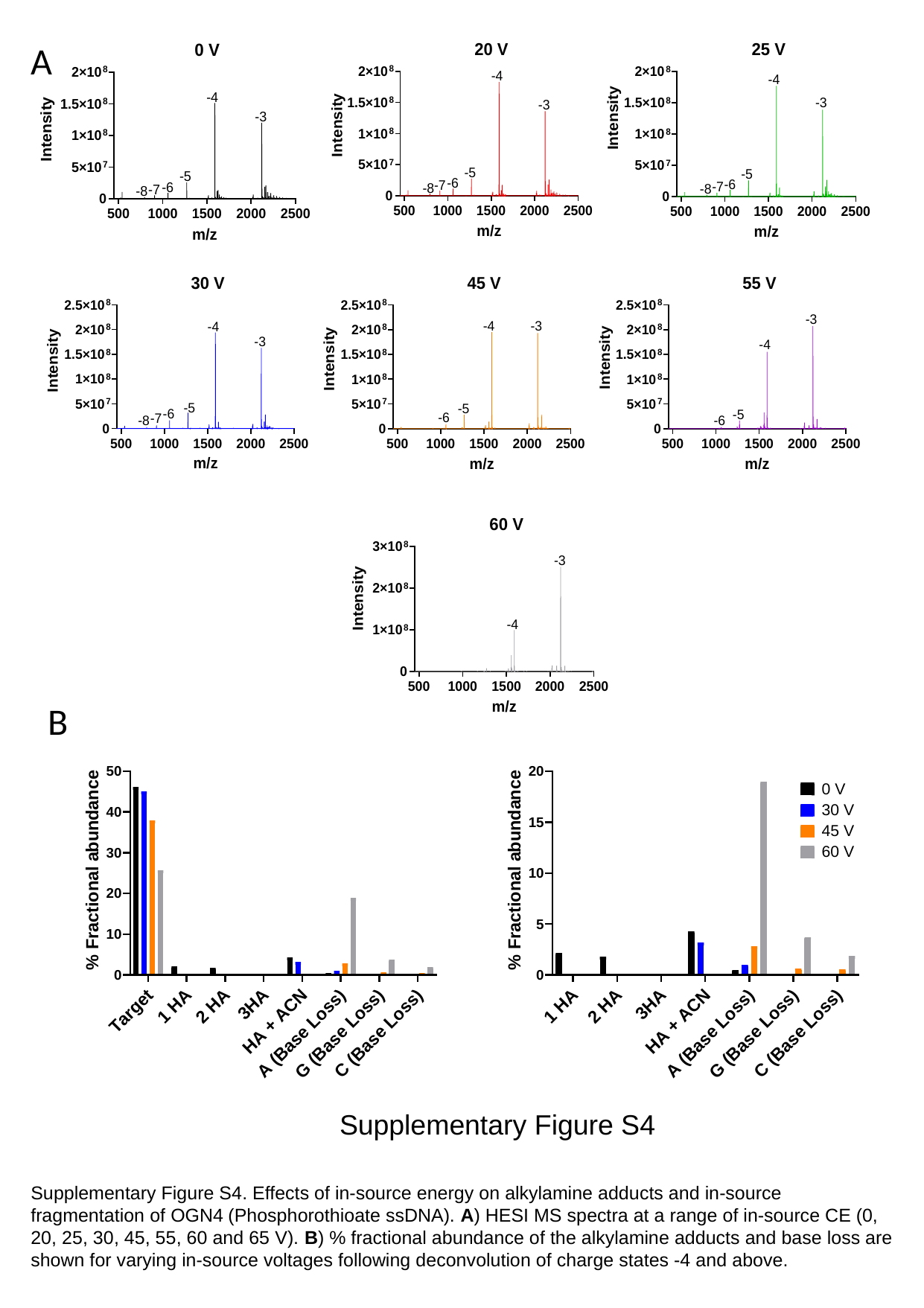

A
B
Supplementary Figure S4
Supplementary Figure S4. Effects of in-source energy on alkylamine adducts and in-source fragmentation of OGN4 (Phosphorothioate ssDNA). A) HESI MS spectra at a range of in-source CE (0, 20, 25, 30, 45, 55, 60 and 65 V). B) % fractional abundance of the alkylamine adducts and base loss are shown for varying in-source voltages following deconvolution of charge states -4 and above.

## Slide 5
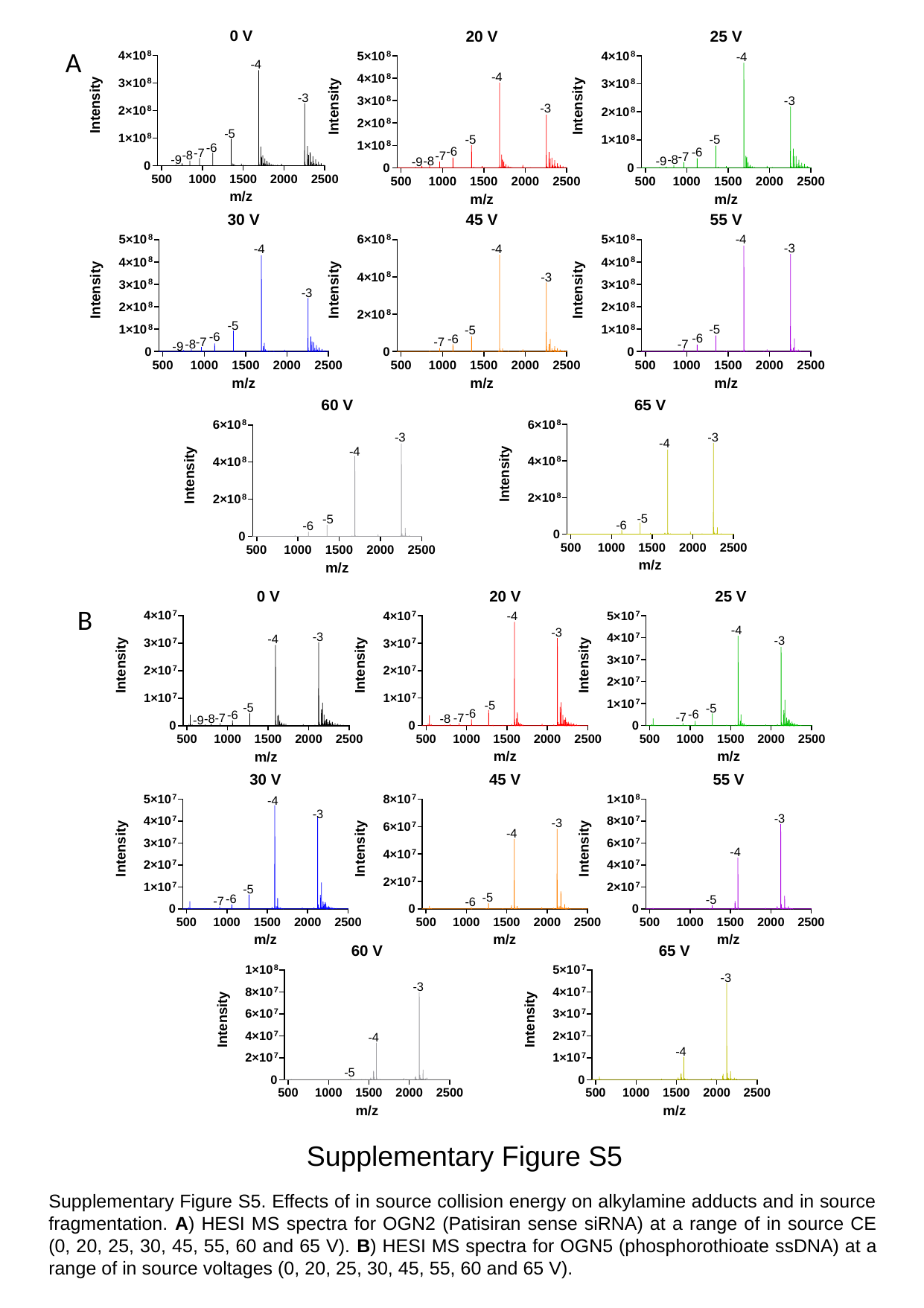

A
B
Supplementary Figure S5
Supplementary Figure S5. Effects of in source collision energy on alkylamine adducts and in source fragmentation. A) HESI MS spectra for OGN2 (Patisiran sense siRNA) at a range of in source CE (0, 20, 25, 30, 45, 55, 60 and 65 V). B) HESI MS spectra for OGN5 (phosphorothioate ssDNA) at a range of in source voltages (0, 20, 25, 30, 45, 55, 60 and 65 V).

## Slide 6
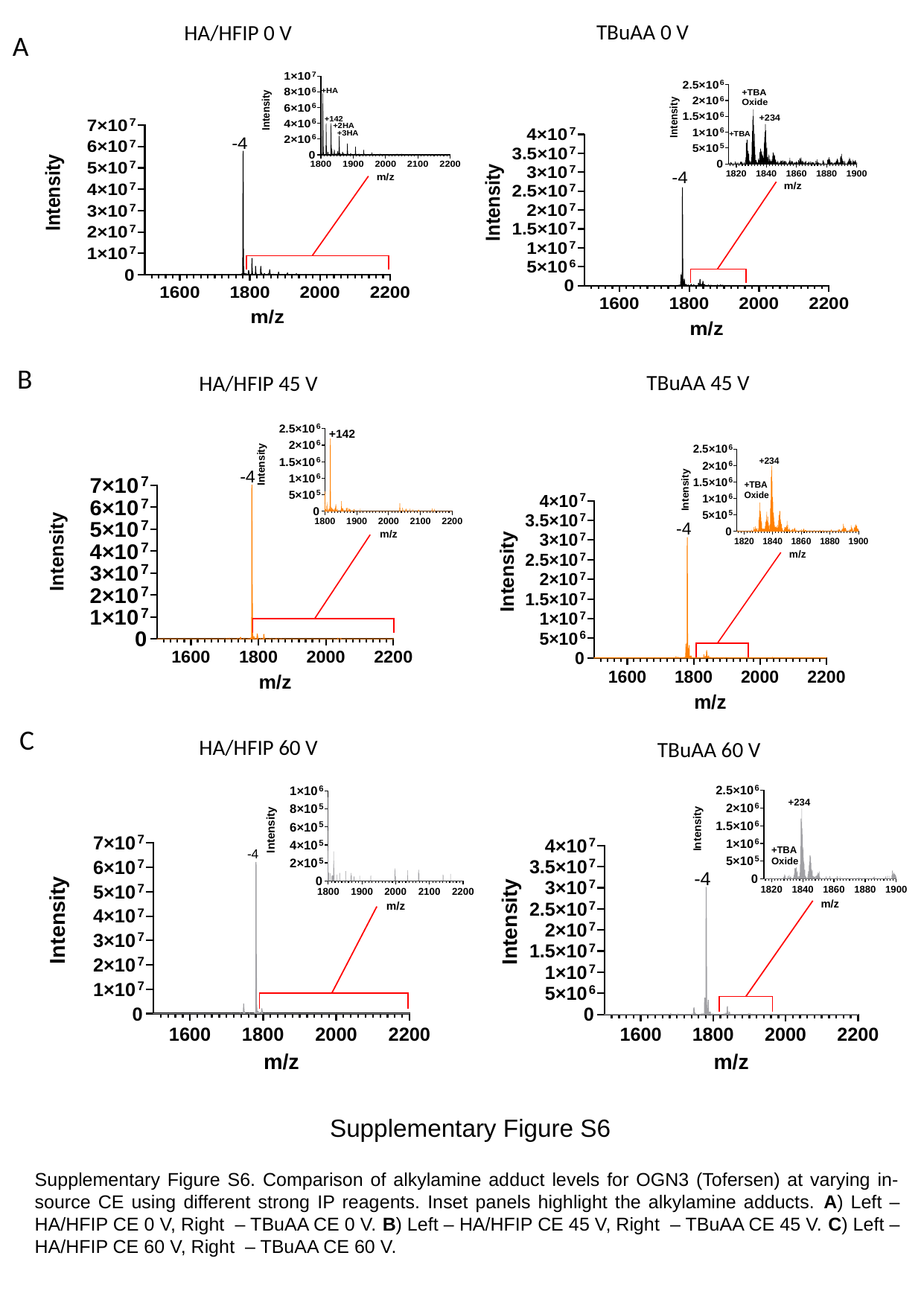

TBuAA 0 V
HA/HFIP 0 V
A
B
C
TBuAA 45 V
HA/HFIP 45 V
HA/HFIP 60 V
TBuAA 60 V
Supplementary Figure S6
Supplementary Figure S6. Comparison of alkylamine adduct levels for OGN3 (Tofersen) at varying in-source CE using different strong IP reagents. Inset panels highlight the alkylamine adducts. A) Left – HA/HFIP CE 0 V, Right – TBuAA CE 0 V. B) Left – HA/HFIP CE 45 V, Right – TBuAA CE 45 V. C) Left – HA/HFIP CE 60 V, Right – TBuAA CE 60 V.

## Slide 7
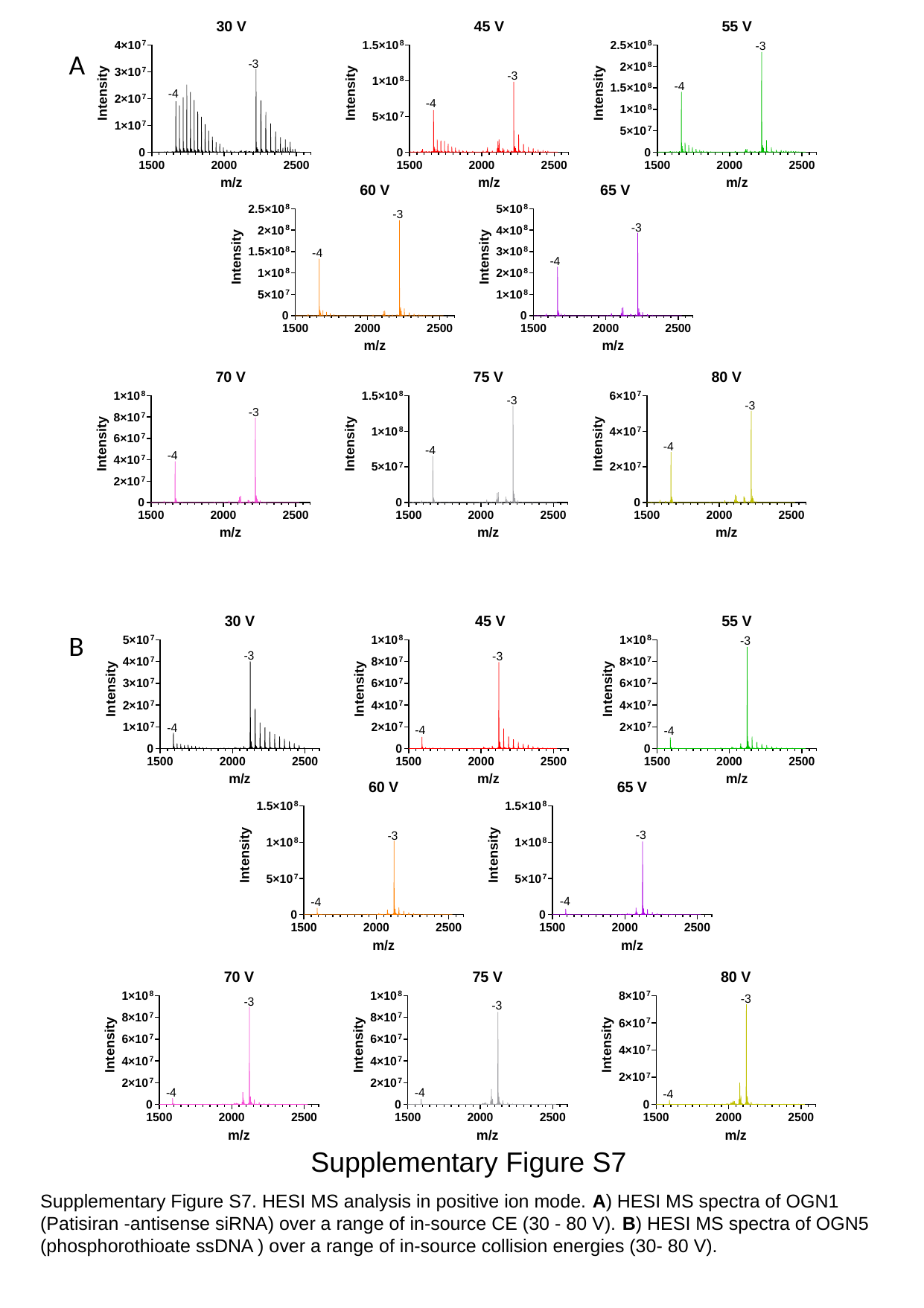

A
B
Supplementary Figure S7
Supplementary Figure S7. HESI MS analysis in positive ion mode. A) HESI MS spectra of OGN1 (Patisiran -antisense siRNA) over a range of in-source CE (30 - 80 V). B) HESI MS spectra of OGN5 (phosphorothioate ssDNA ) over a range of in-source collision energies (30- 80 V).
